# Evaluating sleepiness in operating room settings among anesthesia technicians

**DOI:** 10.1192/j.eurpsy.2025.1876

**Published:** 2025-08-26

**Authors:** M. A. Ghrab, I. Sellami, A. Haddar, A. Feki, M. Hajjaji, M. L. Masmoudi, K. Jmal Hammami

**Affiliations:** 1 Occupational medicine, University Hospital Hedi Chaker; 2 LR/18/ES-28, University of Sfax; 3 Rheumatology, University Hospital Hedi Chaker, Sfax, Tunisia

## Abstract

**Introduction:**

Anesthesia technicians play a crucial role in operating room ensuring the conduct of anesthesia procedures and monitoring patients. However, the demanding nature of their work involving irregular hours, alertness, extended hours in addition to the exposure to anesthetic agents may influence their performance and vigilance.

**Objectives:**

The aim of this study was to assess signs of sleepiness among anesthesia technicians (AT) and evaluate its associated factors.

**Methods:**

We conducted a cross-sectional study among AT in two University Hospitals in Sfax, Tunisia, between January and July 2024 during periodic health assessment visits. Sociodemographic and professional data were collected. The Epworth Sleepiness Scale (ESS) was used to assess signs of sleepiness.

**Results:**

Our population consisted of 60 AT with a mean age of 47.9±7.1 years. Two participants (3.3%) were males. The mean seniority was 24±7.5 years in healthcare and 10.4±8.1 years in the current ward. Forty-five AT (75%) reported using Halogenated Anesthetics. Ninety-five percent of the population had shift work. Fatigue and daytime somnolence were reported by 73.3% and 45% of the population respectively. The median ESS score was 6 Interquartile range IQR [2.25;6]. Excessive sleepiness was found in 21.7% of the population. The ESS score was higher among AT who used halogenated anesthetic, but no significant association was found (p=0.2) in the bivariate analysis.

**Conclusions:**

Assessing vigilance among AT is necessary to maintain patient safety. Organizational factors such as long hours and environmental factors such as effective evacuation system for halogenated agents could cause fatigue and sleepiness in operating room.

**Disclosure of Interest:**

None Declared

